# Enhanced plasmid production in miniaturized high-cell-density cultures of *Escherichia coli* supported with perfluorinated oxygen carrier

**DOI:** 10.1007/s00449-012-0861-7

**Published:** 2012-11-23

**Authors:** Maciej Pilarek, Eva Brand, Friederike Hillig, Mirja Krause, Peter Neubauer

**Affiliations:** 1 Chair of Bioprocess Engineering, Department of Biotechnology, Technische Universität Berlin, Ackerstrasse 71-76, ACK24, 13355 Berlin, Germany; 2 Biotechnology and Bioprocess Engineering Division, Faculty of Chemical and Process Engineering, Warsaw University of Technology, Waryńskiego 1, 00-645 Warsaw, Poland

**Keywords:** High titre plasmid DNA production, *Escherichia coli*, Miniaturized fed-batch culture, Liquid oxygen carrier, Perfluorochemical (perfluorocarbon)

## Abstract

A simple method for plasmid minipreps in closed 1.5 mL microcentrifuge tubes using a cultivation medium with internal substrate delivery (EnBase^®^) in combination with a two-phase perfluorodecalin (PFD) system supplying additional oxygen to the *E.* *coli* culture is described. The procedure can simply be performed on a thermoshaker using only 50 μL cultivation volume. Twenty and twenty-five percent higher cell densities and plasmid concentration, respectively, were obtained with the additional oxygen delivery system when compared to cultures without PFD. Compared to standard 2 mL LB cultures ninefold higher cell densities and eightfold higher plasmid concentrations were achieved for the smaller culture volume. The μL-scale cultures can be directly utilized in further plasmid purification without any centrifugation step or the subsequent removal of the supernatant. This simplifies the routine procedure considerably. Furthermore, the new method is very robust considering the time of cultivation. Highest plasmid concentrations were already obtained after only 6 h of cultivation, but the plasmid concentration remained high (87 % of the maximum) even until 8 h of cultivation. Aside from the advantage of this method for the daily routine, we believe that it could also be applied to automated high-throughput processes.

## Introduction

Successful high-yield isolation of high-quality plasmid DNA is determined by the bacterial cell culture conditions. The aim of all preparative plasmid production cultures should be the selection of best culture conditions to maximize the yield of plasmids in their covalently closed circular DNA (CCC DNA) form. For plasmid purification, well-designed kits are available from many commercial producers. Thus, it is remarkable that the standard cultivation procedure which typically uses 2 mL of LB medium overnight as the standard procedure for miniprep cultures has not been critically evaluated to date in view of robustness and quality control. This is mainly due to the wide need to apply a very simplistic method for plasmid prep from tens or hundreds of samples in the daily routine, a requirement which seems basically fulfilled by the standard method. However, if the handling could be facilitated and at the same time an even better quality control could be incorporated, a newly revised method could be advantageous for the scientific community.

To our knowledge, there have been no detailed investigations on the effects of the overnight cultivation in terms of plasmid quality for plasmid minipreps. However, it is well established in the fermentation literature that the amount and quality of plasmids are affected by nutrient or oxygen limitation [1–3]. Both factors are not well controlled in typical overnight cultures.

We hypothesized that miniaturized μL-scale fed-batch culture systems provide growth control by an enzyme-based glucose delivery method, and are thus similar to production processes for therapeutic plasmid DNA [4–6] would have several advantages: (1) the controlled growth would improve plasmid yield and quality, (2) the enhanced cell density in such fed-batch type cultures would allow a further miniaturization of the culture format. Additionally, the use of standard 1.5 mL microcentrifuge tubes for cultivation would simplify the preparation procedure considerably and also allow consistent controlled conditions in different labs, being a step of implementation of “quality by design” (QbD) to standard biotechnological procedures.

The use of an internal fed-batch cultivation system provided by the EnBase^®^ technology makes the high cell density cultivation of *E*. *coli* possible in small scale and can be performed with readily available media without interruption of the process [7–9]. As significant higher cell densities are reached compared to the standard batch cultures, smaller cultivation volumes can be used. High cell density cultures are usually performed as a carbon-limited fed-batch process by the continuous addition of a highly concentrated carbon source at a growth limiting rate. Importantly, to keep the culture in an aerobic state, the feed rate must be correlated with the oxygen transfer rate. Generally this is limited by the poor solubility of oxygen into the aqueous medium and the mass transfer. Furthermore, the feed rate has a direct influence on the cell growth rate. Consequently, the increase in the volumetric oxygen transfer rate allows a higher substrate feed rate and finally results in higher cell densities or higher metabolite (e.g. plasmids) productivity.

Liquid perfluorochemicals (PFCs) are characterized by a high solubility of respiratory gases (O_2_ and CO_2_) and other non-polar gases. They have raised much interest as fully safe synthetic liquid oxygen carriers in biomedical and bioprocess applications confirmed by many laboratory studies and clinical investigations [10–13]. PFCs are stable and inert compounds with a high resistance to heat sterilization (e.g. by autoclaving) and potential for long-term storage at room temperature [10, 13]. They are immiscible with water and create a separate phase at the bottom of two-phase (water/PFC) systems due to their relatively high density (about 1.9 kg L^−1^) [11, 14, 15]. For biotechnological applications, it is also important that PFCs added to the culture medium do not change the concentration of the medium components (in the case of component concentration calculated per water phase).

In bioprocess applications liquid PFCs are mainly added to culture media as gas carriers and also as scavengers of gaseous byproducts of the cellular metabolism. The oxygen solubility in typical liquid PFCs is 35–44 mM, approximately 20 times higher than the solubility of oxygen in water (2.2 mM) at standard temperature and pressure [14–16]. The solubility of carbon dioxide in liquid PFCs exceeds 200 mM [10, 13, 15]. Many studies have shown that the application of a oxygenated perfluorinated liquids can improve the oxygenation of *E. coli* [17–19] and other microbial [10–13, 20–22], plant cell [10, 11, 23] and animal cell cultures [24–26]. Thereby, this leads to an increased oxygen concentration in the culture medium, and also to improved growth or metabolite biosynthesis in cultures of various kinds of cells. Liquid PFCs could be successfully used as liquid gas carriers especially in μL-scale cultivation systems to prevent oxygen limitation during growth to high cell densities.

The aim of our study was to show that liquid PFCs used as gas carriers can enhance the production of plasmid in miniaturized scale high cell density cultivations. To our knowledge this is the first report of using a PFC in a miniaturized fed-batch culture system to enhance the efficiency of plasmid production, and also one of the first reports on the application of oxygen saturated PFCs in high cell density bacterial cell cultures in general.

## Materials and methods

### Bacterial strain

The recombinant strain of *E. coli* TOP10 carrying the pKS7 plasmid was used in this work. The *E. coli* strain was bought from Invitrogen Co. (USA): OneShot^®^ TOP10 with the following genotype characteristics: [*F* ^*−*^ *mcrA Δ(mrr*-*hsdRMS*-*mcrBC*) ϕ80*lacZΔM15* *ΔlacΧ74 recA1 araD139 Δ(ara*-*leu)* 7697 *galU galK rpsL* (StrR) *endA1 nupG* λ^−^]. The pKS7 plasmid (3,067 kbp) is a Gateway^®^ Entry-vector with a pBR origin carrying and the cloned gene *dgeo 1497*, a purine nucleoside phosphorylase of *Deinococcus geothermalis* and also a kanamycin resistance cassette [27].

### Cultivation medium

EnBase Flo Complex Medium (CM; BioSilta Oy, Finland) was used for the cultivations. The composition of EnBase Flo medium was earlier described by Krause et al. [8]. In this fully soluble culture system glucose is gradually released to the culture from a glucose-containing polymer by a glucoamylase and thereby supporting *E. coli* to grow to high cell densities. EnBase Flo was complemented with Boosting mix (BioSilta Oy, Finland) from the beginning of all cultivations. Kanamycin sulfate stock (15 g L^−1^) was added (1/1,000) to the medium to prevent plasmid loss.

For the reference batch cultures and the inoculum preparation Luria–Bertani (LB) medium was used (10 g L^−1^ Tryptone, 5 g L^−1^ yeast extract and 5 g L^−1^ NaCl; pH was set as 7.0).

### Perfluorinated oxygen carrier

Perfluorodecalin (PFD; C_10_F_18_; 1,1,2,2,3,3,4,4a,5,5,6,6,7,7,8,8,8a-octadecafluorodecalin; ABCR GmbH & Co. KG, Karlsruhe, Germany) was used as a liquid oxygen carrier in this work. PFD was sterilized by autoclavation, cooled to 37 °C. It was then saturated by compressed air and pure oxygen in aseptic conditions supplied with 0.2 μm cartridge filter to prevent microbial contamination as earlier described [11, 19]. The oxygen solubility in PFD does not vary significantly with temperature [28]. In PFD, 4.0 mM O_2_ (0.128 mg mL^−1^) can be dissolved from air [29] or 19.2 mM O_2_ (0.614 mg mL^−1^) from pure oxygen [28] both at 37 °C.

A mixture of 40 % PFD oxygenated with pure O_2_ and 60 % PFD saturated with air was used in all experiments. The total concentration of O_2_ in PFD used has been calculated as 10.08 mM O_2_ (0.322 mg mL^−1^). This oxygen level in PFC was evaluated in our previous studies as providing the best results in the yield of *E. coli* RB791 *pAdh* biomass and the yield of heterologous alcohol dehydrogenase biosynthesis [19, 30].

Cultures not supplemented with PFD were used as a control.

### Cultivation

The *E. coli* TOP10 inoculum was prepared from a pure culture cultivated overnight on a Petri dish with LB medium at 37 °C. The preculture was washed with 1.0 mL of fresh CM and after an OD_600_ measurement the cell suspension was used as an inoculum. All cultures were started with an OD_600_ of 0.15.

Standard 2.0 mL LB medium cultures in a 15 mL bio-reaction tube with loose caps incubated at 37 °C on a shaker with 200 rpm mounted in an approximately 45° angle has been used as reference batch cultures.

The μL-scale cultivations were performed in closed 1.5 mL microcentrifuge tubes (Eppendorf AG, Germany) according to the manufacturer’s protocol for enzymatically controlled fed-batch cultivation systems (EnBase Flo cultivation system; BioSilta Oy, Finland) [8].

The initial culture volume was 50 μL of CM and 50 μL of oxygenated PFD added to the culture at the time of inoculation. Enz I’m glucoamylase (GA; BioSilta Oy, Finland) was also added to all cultures at the time of inoculation to obtain the final concentration of the amylolytic enzyme as 1.5; 3.0; 6.0; 10.0 and 15.0 GAU L^−1^. All parallel cultures were incubated at 37 °C on a thermocycler (Eppendorf AG, Germany) with an orbital shaker (1,400 rpm). The microcentrifuge tubes were tightly closed to prevent oxygen loss due to desorption from the aqueous phase. The aerated culture without any PFD was used as a control.

Parallel cultures were harvested after 4, 6, 8 and 12 h of cultivation to measure the OD_600_ and the pDNA concentration.

### Analytical methods

The cell density was measured spectrophotometrically after the dilution of the culture samples in CM at 600 nm (OD_600_). An OD_600_ value of 1 was equivalent to 1.7 g L^−1^ of wet cell weight and to 0.39 g L^−1^ of dry cell weight of *E. coli* TOP10 biomass.

The concentration of isolated and purified pDNA in each sample (2.0 μL) was directly measured with the ND-1000 UV/Vis spectrophotometer (NanoDrop Technologies, USA) as well as *A*_260_/*A*_280_ and *A*_260_/*A*_230_ ratios. The elution buffer (Tris–EDTA) of the plasmid isolation kit was used as a blank.

### Plasmid isolation and purification

All samples were standardized during the pDNA purification procedure to an equal OD_600_ value of 10. Therefore, plasmid concentration per cell in different samples can be directly compared on the basis of the pDNA concentration.

Plasmid isolation and purification was performed according to the Invisorb^®^ Spin Plasmids Mini Two protocol (Invitek GmbH, Germany). 250 μL of suspension buffer was added directly to 1.5 mL microcentrifuge tubes with the culture samples and vortexed. The next steps of plasmid isolation and purification were proceeding according to the Invisorb^®^ Spin Plasmids Mini Two manual. As the last step, 50 μL of Tris–EDTA elution solution was added directly onto the center of every spin filter surface. Finally, after 1 min of incubation at room temperature, all spin filters were centrifuged for 3 min at 10,000 rpm to eluate the pDNA from the micro-columns according to the manufacturer’s protocol (Invitek GmbH, Germany).

## Results and discussion

### Cell growth

The OD_600_ of *E. coli* TOP10 cells in 2 mL LB medium batch cultures performed in 15 mL bio-reaction tubes after 16 h was found to be 3.44.

**Fig. 1:**
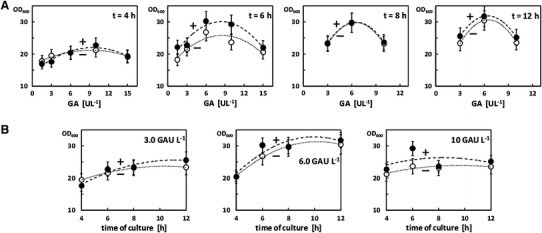
Comparison of the cell density (OD_600_) measured at the same time points of the control (–) and oxygen containing PFD supported (+) miniaturized fed-batch cultures of *E. coli* TOP10 cells. **a** OD_600_ values related to GA concentration in the culture medium at different time points, and **b** growth* curves* related to GA concentration

In all regular experiments the *E. coli* TOP10 cells were cultivated in closed 1.5 mL microcentrifuge tubes filled with 50 μL of CM and additionally supplemented with 50 μL oxygenated PFD (control cultures were devoid of PFD phase). Figure 1 shows the cell density measured in culture samples harvested after 4, 6, 8 and 12 h of culture. The OD_600_ values were generally lower in the control experiments compared to the cultures containing oxygenated PFD. However, differences did not exceed 20 %. The highest cell density (OD_600_ = 31.8) was obtained in the sample harvested after 12 h of cultivation in the presence of oxygen enriched PFD at a concentration of 6.0 U L^−1^ of GA. Therefore the highest value of OD_600_ obtained for the PFD supported μL-scale fed-batch cultures was ninefold higher cell density compared to the reference batch culture.

### Plasmid production

We have studied the production of plasmid using TOP10 *E. coli* cells carrying the pKS7 plasmid as an example. The average value of plasmid concentration in the 2 mL LB medium batch reference cultures performed in 15 mL bio-reaction tubes was 3.1 μg mL^−1^.

In standard procedures for plasmid isolation in mL-scale cultivation systems, the culture must be centrifuged and the supernatant discarded prior to the addition of the suspension buffer to the tubes containing the pelleted cells. Liquid PFCs are chemically inert, they are immiscible with aqueous media and they create a separate phase in PFC/aqueous systems in the bottom. Thus, the PFD did not have any influence on the plasmid analysis procedure; the plasmid isolation can be continued directly in the same 1.5 mL microcentrifuge tube that was used for the cultivation.

**Fig. 2:**
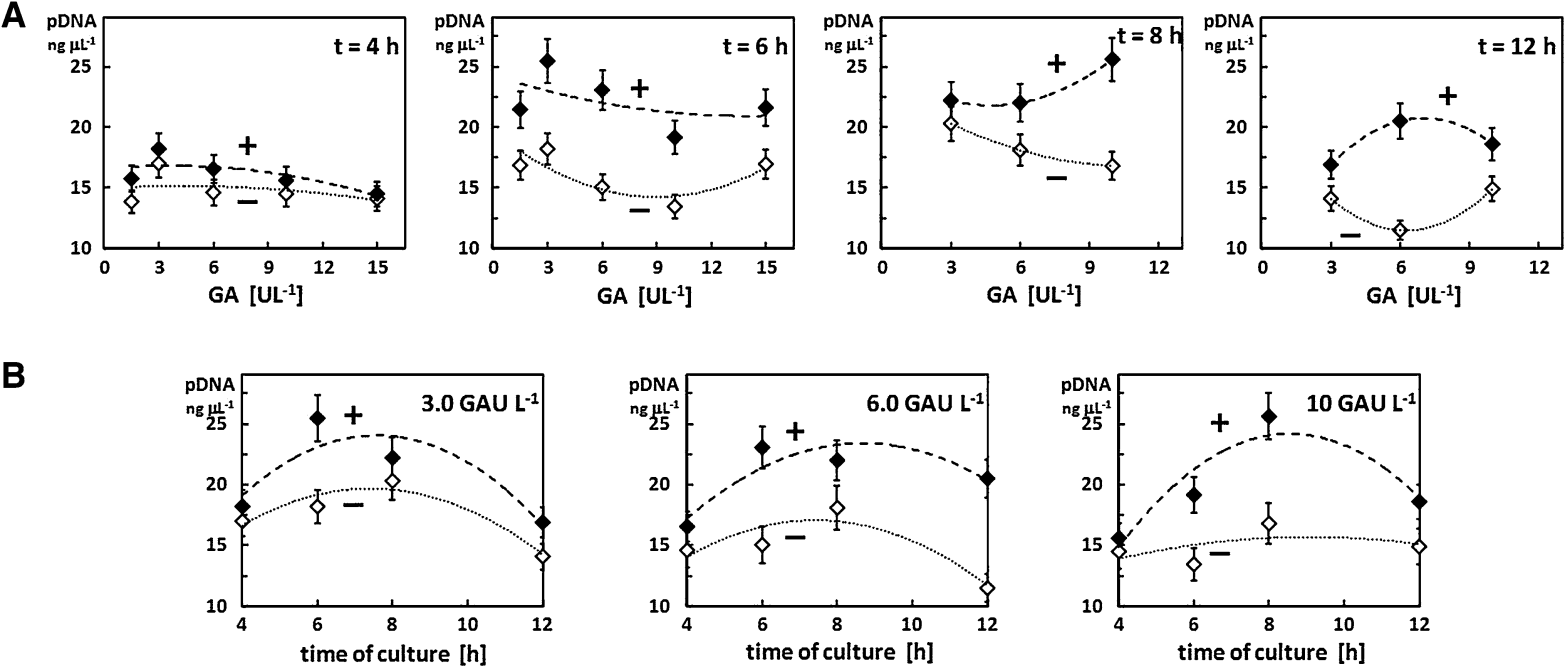
Comparison of the pKS7 plasmid (pDNA) concentration (range of ng μL^−1^) measured spectrophotometrically in the standardized (OD_600_ value of 10) samples of *E. coli* TOP10 cells at the same time-points of the control (–) and the oxygenated PFD supported (+) miniaturized 50 μL fed-batch cultures. **a** Plasmid concentration related to GA concentration at different time points, and **b** dynamics of the plasmid concentration over the cultivation time at different GA concentrations

**Fig. 3:**
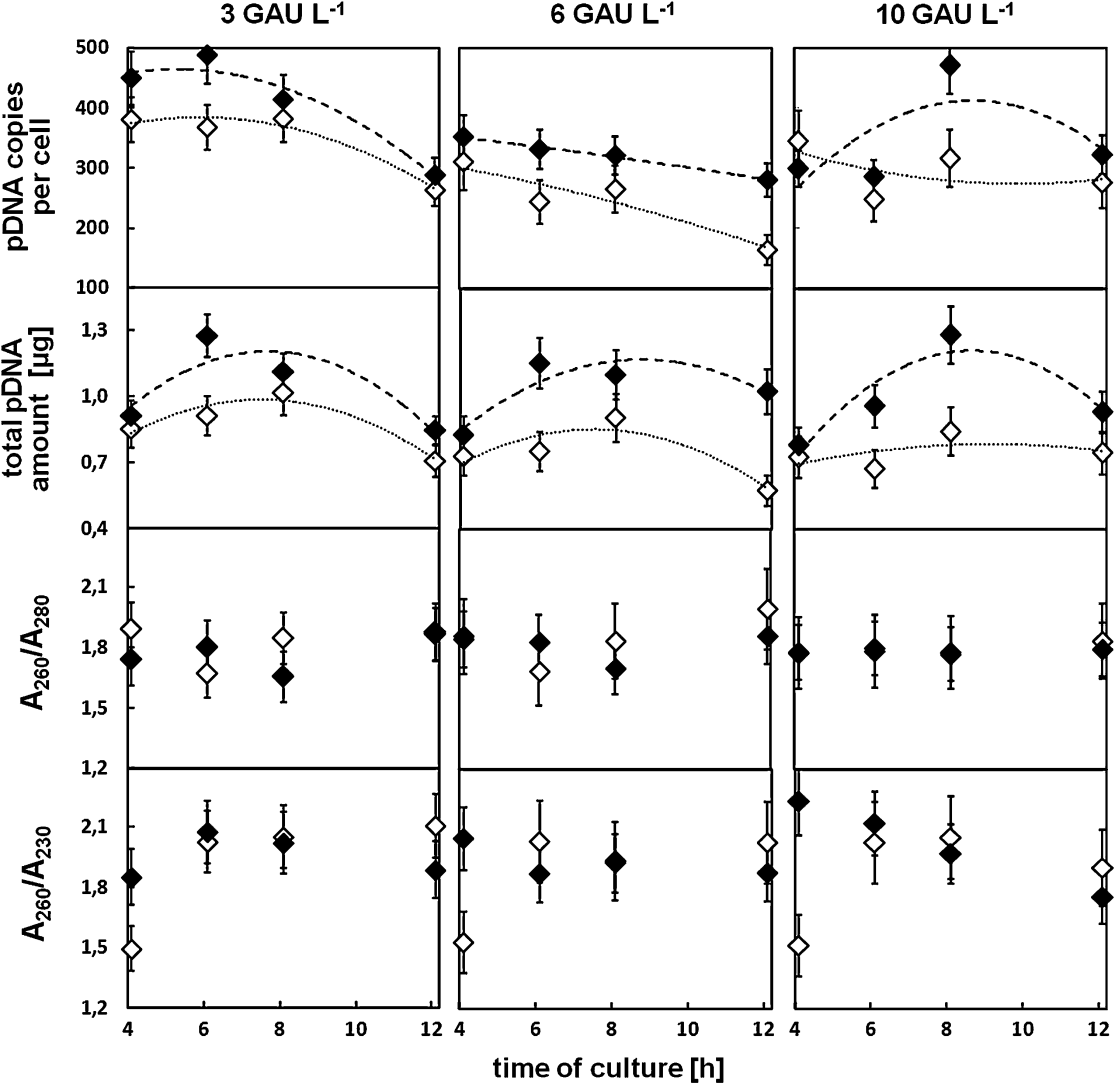
Concentration and purity of pKS7 plasmid isolated from parallel cultures supported with PFD (*black filled marks*) and control cultures without PFD (*white filled marks*): **a** plasmid copies per cell, **b** total plasmid amount obtained from single culture, **c** values of the *A*_260_/*A*_280_ ratio and **d** *A*_260_/*A*_230_ ratio obtained in spectrophotometric analysis of isolated pDNA

The pKS7 plasmid is generally produced at high concentrations. However, small changes in culture conditions can affect the pDNA biosynthesis, i.e. the plasmid copy number per cell. We aimed to see whether the oxygen containing PFD supplementation would result in a higher plasmid copy number per cell and thus in a higher plasmid concentration per culture. The pDNA concentration in standardized (OD_600_ value of 10) samples of the *E. coli* TOP10 cells which were cultivated with and without PFD in 1.5 mL microcentrifuge tubes are presented in Fig. 2. High concentrations of plasmid were seen in the cultures containing PFD and a concentration of amylolytic enzyme in a wide range from 3.0 to 10.0 GAU L^−1^. These plasmid concentrations were significantly higher compared to the control cultures without PFD (Fig. 2). Higher plasmid copy numbers per cell have been obtained in every case of PFD supported cultures compared to the control cultures (Fig. 3a). The number of plasmid copies per cell seems to correlate with the cultivation time. Significantly higher values have been noted for shorter cultivation times than for longer ones (cf. Fig. 3a). Also the total amount of isolated plasmid obtained from a single culture was higher in the case of cultures with PFD than without the additive (Fig. 3b). The purity levels of isolated plasmid were similar in both culture systems (they are presented as *A*_260_/*A*_280_ and *A*_260_/*A*_230_ ratios on Fig. 3c and d). The highest total amount of plasmid isolated from a single culture (i.e. 1.27 μg of pDNA isolated from one 50 μL culture) was obtained after 6 h of cultivation in the presence of PFD at a concentration of 3.0 U L^−1^ GA. Here the plasmid level was about 40 % higher than in the equivalent control culture, and 21 % higher than the highest plasmid level observed in all the studied control cultures without PFD. Looking at plasmid concentration the highest value obtained for the PFD supported μL-scale fed-batch system (25.45 ng μL^−1^) was eightfold increased compared to the reference batch cultures. Also interestingly, the measured plasmid concentrations correlated both with the concentration of added amylolytic enzyme (cf. Fig. 2a). In the control cultures without the PFD the plasmid concentration remained high over a longer cultivation period and reached its maximum value after 8 h in almost all cases. In the PFD supplemented cultures the maximal value was seen earlier, i.e. after 6 h of cultivation, after which the plasmid concentration decreased slightly.

The common procedure for plasmid production which is routinely used in molecular biology laboratories includes a 2 mL overnight batch LB or NB medium cultivation in 10–15 mL bio-reaction tubes. This is followed by plasmid isolation with one of the commercial kits with centrifugation of the cells and finally, the re-suspension of the pellet in lysis buffer as the first steps of the following purification procedure. The major aim of this study was to investigate possible benefits of the miniaturized fed-batch technique and PFC-based gas carriers in μL-scale cultures of bacterial cells. The PFC-based cultivation μL-system is easy to apply and could be performed very simply with regular laboratory devices, i.e. in thermoshakers. A significant advantage of the use of closed microcentrifuge tubes applied in our studies is the prevention of evaporation of medium which is a severe problem in small scale cultivation systems. Our results explicitly indicate that miniaturized fed-batch cultures realized in closed 1.5 mL microcentrifuge tubes containing oxygenated PFC provide a significant improvement of the efficiency of plasmid biosynthesis in *E.* *coli* cells. The plasmid copy number per cell was improved by up to 28 % and the total amount of plasmid obtained from a single culture was improved up to 21 % compared to the cultures not supplemented with PFD (cf. Fig. 3b). Simultaneously, the differences in the level of cell density were not as high as it was previously reported for *E. coli* cell cultivated in the mL-scale deep-well cultures [19, 30]. Nevertheless, a 10–20 % higher cell density was noted in all PFD supplemented cultures in the present study compared to the control cultures (cf. Fig. 1). Our results show that supplementation of the cultures with oxygenated PFD makes the plasmid biosynthesis much more robust and has a positive effect on the total pDNA concentration obtained.

Supplementation of L-scale bacterial cultures with oxygenated PFD has been previously applied to increase the oxygen transfer [10, 12, 14, 21]. The high costs of PFCs and minor improvement of growth in some cases are the main reasons why PFCs are only rarely applied in the field of experimental biotechnology. In μL-scale cultures the costs for PFC are insignificant due to the small volumes.

Some studies clearly discuss the influence of oxygen limiting conditions on plasmid productivity in miniaturized *E. coli* cell cultures [31, 32], even though they just present plasmid yields in cultures carried out under anaerobic, oxygen limited or aerobic conditions. Oxygen limitations neither seems to have a direct negative influence on plasmid stability nor the plasmid copy number, however, the growth of *E. coli* to high cell densities is negatively influenced under oxygen limitation by the low biomass yield per glucose and the acidification of the culture medium by the mixed acids fermentation. In view of plasmid production processes, many studies clearly prove that *E. coli* fed-batch cultures provide much higher plasmid yields than batch processes [5, 31–35]. Based on the data presented in Table 1 the advantages of the PFD-supported μL-scale fed-batch culture (i.e. high concentration of pDNA obtained after only 6 h, and with only 50 μL of medium) over other approaches in mL- and L-scale cultures carried out over night or for up to 24 h, can be easily demonstrated.

**Table 1:** Comparison of high titre plasmid DNA cultures of *E. coli* cells under a range of different culture conditions

Type of culture	*E. coli* strain (type of plasmid)	Culture medium	Culture volume	Max. OD_600_	pDNA (mg L^−1^)	Culture time (h)	References
Batch (bioreactor)	DH5α (pSVβ)	LB	10 mL	20–25	3.5–4.0	10	Betts et al. [31]
Batch (bioreactor)	DH5α (pSVβ)	LB	7 L	20–25	3.5–4.0	10	[31]
Batch	DH5α (pEGFP-N1)	LB	3 mL	2.5	<7.0	16 (overnight)	O’Mahony et al. [32]
Batch	DH5α (pEGFP-N1)	LB + glycogen	5 mL	3.7	<7.0	16 (overnight)	[32]
Batch	DH5α (pEGFP-N1)	LB + complex additives	5 mL	8.5	7.0	16 (overnight)	[32]
Fed-batch (bioreactor)	DH5α (pEGFP-N1)	Complex medium	10 L	50	20	20	[32]
Fed-batch (bioreactor)	DH5α (pEGFP-N1)	Complex medium	3 L	80	250	20	[32]
Fed-batch (bioreactor)	DH10B (pVCL1005)	LB + glycerin	10 L	56.5	220	21.5	Lahijani et al. [33]
Fed-batch (bioreactor)	DH10B (pENV)	Complex medium	80 L	120	100	24	Chen et al. [34]
Batch	DH5α (pSVβ)	LB + additives + glucose	2 L	4	1.25	12	O’Kennedy et al. [5]
Batch (bioreactor)	DH5α (pSVβ)	LB + additives + glucose	5 L	7	8.5	24	[5]
Fed-batch (bioreactor)	DH5α (pSVβ)	LB + additives + glucose	5 L	15	29.7	24	[5]
Fed-batch (μL-scale)	OneShot^®^ TOP10 (pKS7)	EnBase^®^-Flo complex medium	50 μL	30	25.45	6	Results presented in current paper

## Conclusions

The results of this study have shown the practicability of plasmid purification from only 50 μL *E. coli* cell cultures cultivated in closed standard 1.5 mL microcentrifuge tubes on a regular thermomixer. It was shown that it is possible to obtain over 1 μg of plasmid per culture. The advantages of this method are: (1) the reproducible and fast cultivation (within 6–8 h), (2) the use of regular laboratory devices without the requirement for an additional shaker, (3) the closed microcentrifuge tube prevents evaporation of the medium, (4) the addition of the oxygen enriched liquid gas carrier (i.e. PFD) impedes the culture from oxygen limitation and finally (5) the possibility of continuing the in situ plasmid isolation directly in the 1.5 mL microcentrifuge tubes without centrifugation; the same tubes where the cultures were grown, which simplifies and decreases labor and time of the whole plasmid preparation process. In summary the method shortens cultivation time to 6–8 h, which allows plasmid preparation within 1 day. Our results indicate that the PFD oxygenation system is valuable in miniaturized cell-culture formats for plasmid production. It may be suggested that the method would also offer a benefit to the automation of plasmid preparations.
